# Under pressure—the influence of hypergravity on electrocortical activity and neurocognitive performance

**DOI:** 10.1007/s00221-023-06677-8

**Published:** 2023-08-04

**Authors:** Constance Badalì, Petra Wollseiffen, Stefan Schneider

**Affiliations:** 1 Institute of Movement and Neurosciences, German Sport University Cologne, Am Sportpark Müngersdorf 6, 50933 Cologne, Germany; 2 Centre for Health and Integrative Physiology in Space (CHIPS), German Sport University Cologne, Cologne, Germany

**Keywords:** Hypergravity, Parabolic flight, Brain activity, Behavioural and neuronal parameters, ERP, EEG

## Abstract

The effects of hypergravity and the associated increased pressure on the human body have not yet been studied in detail, but are of great importance for the safety of astronauts on space missions and could have a long-term impact on rehabilitation strategies for neurological patients. Considering the plans of international space agencies with the exploration of Mars and Moon, it is important to explore the effects of both extremes, weightlessness and hypergravity. During parabolic flights, a flight manoeuvre that artificially creates weightlessness and hypergravity, electrocortical activity as well as behavioural parameters (error rate and reaction time) and neuronal parameters (event-related potentials P300 and N200) were examined with an electroencephalogram. Thirteen participants solved a neurocognitive task (mental arithmetic task as a primary task and oddball paradigm as a secondary task) within normal as well as hypergravity condition in fifteen consecutive parabolas for 22 s each. No changes between the different gravity levels could be observed for the behavioural parameters and cortical current density. A significantly lower P300 amplitude was observed in 1 G, triggered by the primary task and the target sound of the oddball paradigm. The N200, provoked by the sounds of the oddball paradigm, revealed a higher amplitude in 1.8 G. A model established by Kohn et al. (2018) describing changes in neural communication with decreasing gravity can be used here as an explanatory approach. The fluid shift increases the intracranial pressure, decreases membrane viscosity and influences the open state probability of ion channels. This leads to an increase in the resting membrane potential, and the threshold for triggering an action potential can be reached more easily. The question now arises whether the observed changes are linear or whether they depend on a specific threshold.

## Introduction

The spirit of adventure and discovery has always driven mankind to further explore space and push the limits of what is possible in extreme environments, as, for example, environments with reduced or increased gravity. Understanding the effects of these extremes on the human body, including the central and peripheral nervous system, is particularly relevant.

In the last decades, several studies have investigated human behaviour and neurocognitive performance in microgravity, e.g. during space flights or experimental flights, which could be a parabolic flight where hypergravity and weightlessness are created artificially (Koppelmans et al. 2013; Kozlovskaya et al. 1981; Wollseiffen et al. 2019, 2016). There is, on the one hand, evidence that cognitive functions are impaired during space flights, such as central postural functions (Kozlovskaya et al. 1981) or attentional processes (Pattyn et al. 2005). This is not surprising, as the human body is used to an environment with permanent gravity throughout motor and cognitive development and has adapted accordingly (Kozlovskaya et al. 1981). However, more recent studies could show lower cortical activity in 0 G compared to 1 G during short periods of weightlessness (Klein et al. 2019), which is related to the results of Wollseiffen et al. (2019), which presented lower amplitudes of event-related parameters in combination with significantly reduced reaction time in weightlessness whilst maintaining the same error rate. These results suggest that both neural processes and behavioural markers benefit from an environment with reduced gravity (Wollseiffen et al. 2019). Both authors assume an increased intracranial pressure, caused by a blood redistribution during weightlessness, as an explanation (Lawley et al. 2017). This is consistent with the physiological model developed by Kohn and Ritzmann (2018), which was derived from in vivo and in vitro data and indicates a slightly depolarized resting membrane potential of neurons under weightlessness (Kohn and Ritzmann 2018).

Hypergravity, at this point in time, has not yet been intensively researched with regard to cognitive function. Even though, some studies could show that phases of hypergravity have an effect on the human body. Smith et al. (2013) reported increased cortical activity from artificially induced higher gravity levels on a short arm human centrifuge (Marušič et al. 2014; Smith et al. 2013), whereas Schneider et al. (2008) showed lower activity of various frequencies in hypergravity compared to normal gravity.

These phases of hypergravity occur mainly during space flights, especially throughout take-off and landing manoeuvres. Therefore, the findings from hypergravity and weightlessness in combination should ensure the safety of the astronauts during space missions. Furthermore, it should allow an insight into the physiological changes caused by different gravity levels to positively influence the rehabilitation measures for neurological patients in the long term.

The aim of this study was to investigate a possible influence of hypergravity on electrocortical activity and to quantify its effects on neurocognitive performance. Based on the model of Kohn and Ritzmann (2018) and the results already found in microgravity by Klein et al. (2019) and Wollseiffen et al. (2019), we hypothesise that hypergravity may lead to an increased electrocortical activity compared to normal gravity and affect the behavioural performance.

## Materials and methods

### Participants and procedure

Parabolic flights led by the European Space Agency (ESA) and the German Space Agency (DLR) take place from Merignac International Airport in Bordeaux (F) on board the A310 ZeroG. One parabola consists of four different flight phases and is characterised by gravity changes from 1 G to 1.8 G, to 0 G, back to 1.8 G and finally again to 1 G. One flight campaign consists of 3 flight days, with 30 experimental parabolas completed on each flight day. During two campaigns, which took place between November 2020 and October 2022, data were recorded from 13 participants (6 male, 7 female) who performed the experiment in 1 G as well as in the first 1.8 G phase of a parabolic manoeuvre. All participants and investigators were clinically examined beforehand and gave informed consent. Prior, the experimental design of the study was approved by the Research Ethics Committee of the German Sport University Cologne and University of Caen in accordance with the Declaration of Helsinki.

### Experiment

The neurocognitive task consisted of a classical auditive oddball paradigm in combination with a mental arithmetic task, which had to be solved simultaneously. The task has been described before in detail (Wollseiffen et al. 2019). Briefly: during the oddball paradigm, the participants heard, in a randomised order via noise-cancelling earphones (BOSE quite Comfort 30), either target sounds (high pitch tones) or standard sounds (low pitch tones). The low tones (70% of all tones, standard) had to be ignored by the participant; however, if a high pitch tone occurs (30% of all tones, target), the participant had to react and press the space bar on a keyboard on their lap as quickly as possible. At the same time, the participants were presented with a mental arithmetic task. They had to decide between two equations (e.g. ‘(7 − 4) × 3’ vs. ‘4 + 4’), displayed on the left and right side of a 27-inch iMac screen, which yields the larger result.

**Fig. 1:**
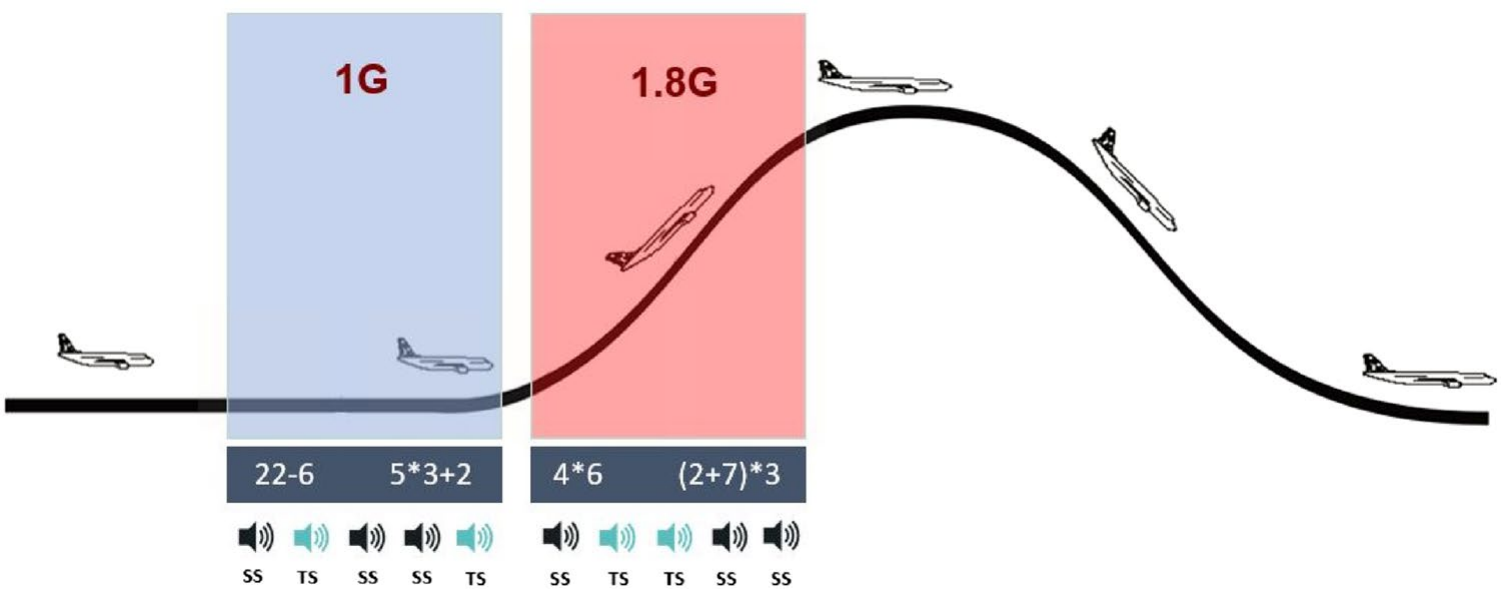
Graphical representation of the neurocognitive task, which consisted of a mental arithmetic task and a classical oddball paradigm. Participants had to decide between two equations which yields the larger result and indicate it by pressing the right or left arrow key on a keyboard. Simultaneously, if a high pitch tone occurred, participants had to press the space bar on the keyboard. The task had to be solved during the 1 G and 1.8 G phase in 15 consecutive parabolas on 1 flight day. *SS* standard sound, *TS* target sound

The participants had to press the right or left arrow key on the keyboard as quickly as possible, according to their answer. The appearance of the mental arithmetic task was dependent on the participants’ reaction to the task. To minimise the reaction time of the participants, their fingers were resting on top of the keyboard during the entire time of the experiment. A schematic representation of the experiment is depicted in Fig. 1.

The participants were instructed to concentrate primarily on the mental arithmetic task and to solve it as quickly and accurately as possible, and secondarily on the sounds of the oddball paradigm. The participants worked on the task during 15 consecutive parabolas on 1 flight day. All participants were given an anti-nausea drug (scopolamine) in the morning before the flight, which has been shown not to impair neurocognitive performance (Wollseiffen et al. 2016). During the entire flight, participants were securely attached to the floor of the aircraft with a strap to avoid uncontrolled flying and loss of orientation on the one hand, but also allowed participants to experience the sensation of reduced gravity on the other hand. In addition, participants were observed by an operator to avoid outside disturbances and to reassure them during periods of altered gravity. All participants went through the experimental protocol 24 h before the start as a familiarisation process.

### EEG data collection

Each participant wore an EEG cap (actiCap-32Ch, Brain Products GmbH, Munich, Germany) fitted to his or her individual head circumference and arranged in the classic 10–20 configuration. Each electrode was referenced to a reference electrode located in the triangle of FP1, FP2 and Fz. The ground electrode was located directly next to it. For optimal signal transmission, all electrodes were filled with Electro-Gel™ (Electro-Cap International, USA). To keep the impedance below 10 kΩ during the flight, the electrodes were regularly refilled with gel. Before storing the EEG recording, the analogue data were amplified and converted into digital signals via Brain Vision amplifier and RecView software (Brain Products GmbH, Munich, Germany).

### EEG data analysis

The EEG signals were analysed offline using Brain Vision Analyzer 2.2 (Brain Products, Munich, Germany). After applying low-pass and high-pass filters, a frequency range between 3 and 40 Hz remained for the analyses (Time constant 0.531 s; 48 dB/octave). Individual channels with an impedance of more than 10 kΩ were interpolated (order of splines: 4, maximum degree of Legendre polynomials: 10, standard lambda: 1E-05). After visual inspection, eye correction was performed using independent component analysis to identify and eliminate blinks and horizontal eye movements.

### Current cortical density

After primary segmentation into 1 G and 1.8 G and secondary into 4 s intervals, an automatic artefact rejection was performed (gradient < 50 μV; max/min amplitude −200 to 200 μV; lowest allowed activity in intervals 0.5 μV). Since the recorded scalp potentials have different volumes depending on the localization of the reference, data for each participant were transformed into reference-free current source density (CSD) maps (order of splines: 4; maximum degree of Legendre polynomials: 10; lambda 1e-5). The CSD takes the voltage values of the individual electrodes with the current source density at these electrodes. Using the integrated LORETA (Grech et al. 2008; Pascual-Marqui 2002) module in the Brain Vision Analyzer, cortical current densities in the frontal, parietal, occipital lobes and the region supplied by the middle cerebral artery (MCA) were determined across each 4 s recording interval. Cortical current density defines the electrical current caused by neural activity per unit area of cross-section. In general, the unit is microvolts per square millimetre (electrical current in a 2-dimensional area) but in a voxel-based analysis, this value needs to be squared so that the unit is squared microvolts per millimetre to the power of 4.

### Event-related potentials

Event-related potentials not only describe the perception and processing of sensory information, but also represent higher cognitive processes (Duncan et al. 2009). Two specific ERP components, N200 and P300, occur primarily in behavioural control processes, encompassing the evaluation of stimuli, selective attention and conscious perception, amongst other aspects (Helfrich and Knight 2019; Patel and Azzam 2005). ERPs are, in contrast to spontaneous brain activity, potential shifts that occur after a specific stimulus, which can be of a sensoric, motor or psychic nature (Deetjen et al. 2005). Due to their significantly lower amplitude (approximately 10 µV), ERPs are often superimposed by the higher spontaneous EEG (up to 100 µV). Since ERPs occur time-specifically, we identified the appearance of the task (i.e. the display of numbers on the screen as well as target and standard sound) as the relevant stimuli for the segmentation. After this segmentation based on stimulus onset (−200 to 800 ms), an automatic artefact rejection was applied (gradient < 50 μV; max/ min amplitude −200 to 200 μV; lowest allowed activity in intervals 0.5 μV) and the data were baseline corrected (−200 to 0 ms). Data were then averaged for each participant for the 1 G and the 1.8 G condition over all electrodes. All ERP waveforms were converted into reference-free CSD maps (order of splines: 4, maximum degree of Legendre polynomials: 10, standard lambda: 1e-5). Peak detection algorithms were applied to check the peak position, amplitude and latency of the respective ERPs.

### Statistics

Statistical analysis was performed using R (2022.12.0, Posit Software, PBC). All data were checked for normality using the Shapiro–Wilk normality test beforehand. The LORETA data were analysed using a repeated measure ANOVA or the non-parametric version, the ld.f2 function with the Wald-type (WTS) of the *nparLD* package in R, with the within-group factor *gravity* (1 G/1.8 G) and *region of interest* (ROI) (frontal, parietal, occipital and region supplied by the MCA). If necessary, a pairwise *t *test/Wilcoxon test including the Bonferroni correction was used as a post hoc test.

Regarding the performance of the participants, error rate and reaction time were analysed using either a repeated measure ANOVA or Friedman test with the within-group factor *gravity* (1 G/1.8 G) and *task* (arithmetic task/target sound/standard sound). If necessary, a pairwise *t* test/Wilcoxon test including the Bonferroni correction was used as a post hoc test.

For the statistical analysis of the amplitude and latency of the ERPs N200 and P300, data were resampled using the Jackknife resampling approach to estimate the variance and bias that might occur (Efron and Stein 1981). Either a *t* test or Wilcoxon test was used to calculate differences of the amplitude and latency of the event-related potential P300 regarding the gravity levels.

The same parameters for the ERP N200 revealed a non-parametric distribution of the data following an outlier in one group. This is not uncommon, especially in human physiological experiments, but should be included for further analysis. Since this study was performed with a small sample size and the observation of an outlier, the non-parametric version of a two-factorial repeated measure ANOVA, the ld.f2 function with the Wald-type (WTS) of the *nparLD* package in R, was used, with the within-group factors *gravity* (1 G/1.8 G) and *task* (target sound/standard sound) (Brunner and Langer 1998; Feys 2016; Noguchi et al. 2012). This package refers to results of the study by Akritas and Brunner (1997). Here, relative treatment effects are defined in relation to the distributions of the variables measured in the experiment. If necessary, a pairwise *t* test or Wilcoxon test including the Bonferroni correction was used as a post hoc test.

The level of significance was set to *p* < 0.05. Data in this manuscript are presented as mean and standard deviation.

## Results

**Fig. 2:**
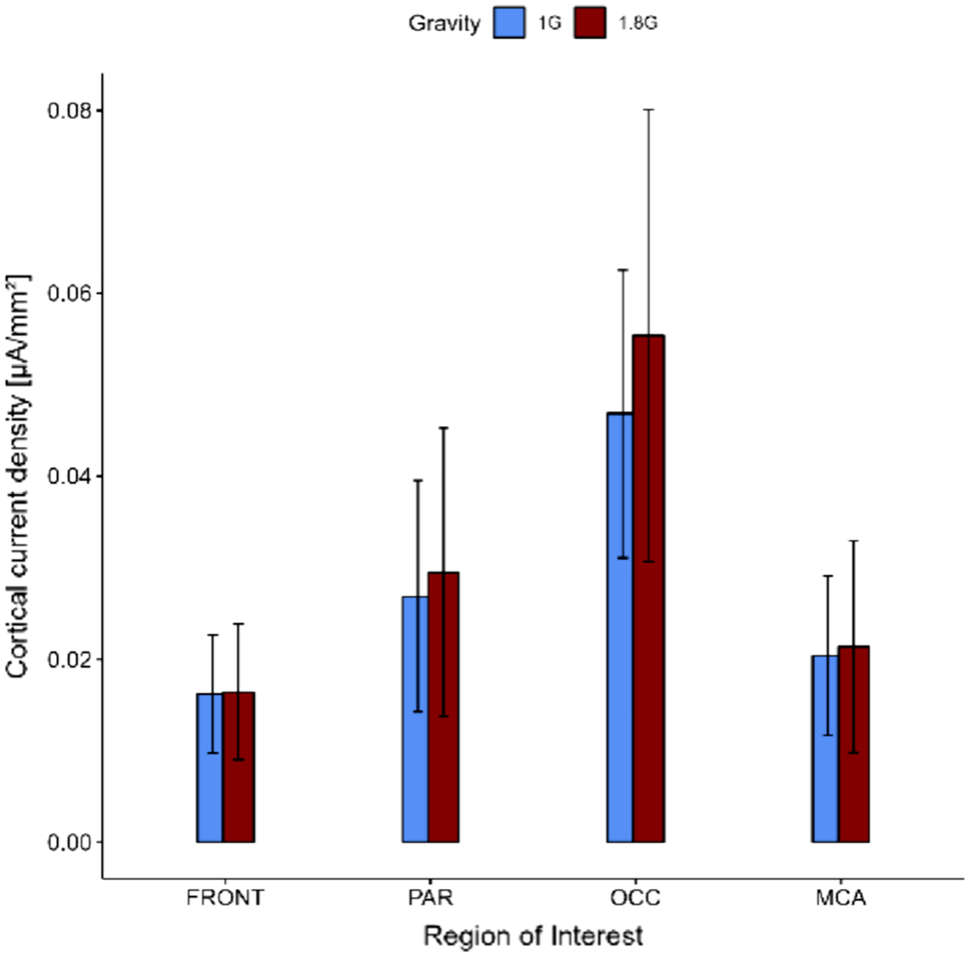
Cortical current density over 15 trials for 22 s of the neurocognitive task in 1 G (blue) and 1.8 G (red). Displayed are means ± standard deviation. *FRONT* frontal lobe, *PAR* parietal lobe, *OCC* occipital lobe, *MCA* middle cerebral artery

**Fig. 3:**
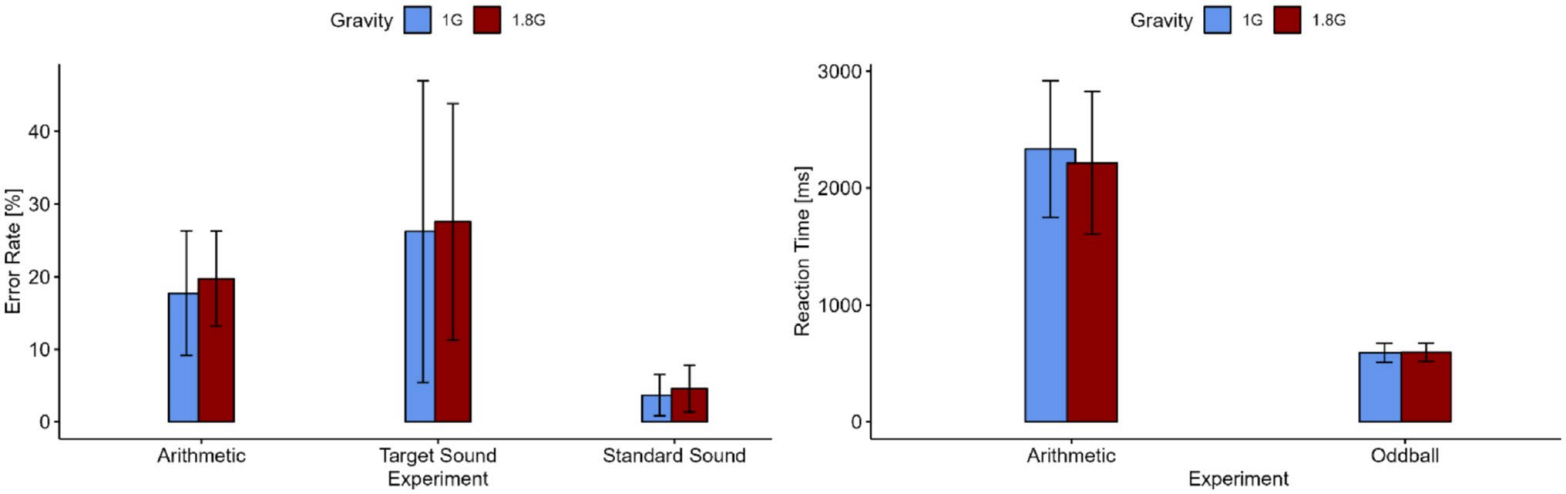
Error rate (left) of the answers for the mental arithmetic task as well as the target and standard sound for the oddball paradigm in 1 G (blue) and 1.8 G (red). Reaction time (right) for the mental arithmetic task and the target sound of the oddball in 1 G (blue) and 1.8 G (red). Displayed are means ± standard deviation

Results of the ld.f2 model with the Wald type (WTS) showed no interaction effect of *ROI* and *gravity* for electrocortical activity (Statistic = 4.031, *p* = 0.258) but a main effect of the factor *ROI* (Statistic = 201.849, *p* < 0.001) (s. Fig. 2; Table 1). With regard to the performance of the participants, the repeated measure ANOVA showed no significant interaction effect of the factors *gravity* and *task* for reaction time (*F*_(1.00,12.00)_ = 4.34, *p* = 0.06) and error rate (*F*_(2.00,24.00)_ = 0.06, *p* = 0.94) (s. Fig. 3; Table 2).

**Table 1:** Cortical current density of 13 participants during normal gravity (1 G) and hypergravity (1.8 G)

	Cortical current density [μA/mm^2^]			
	FRONT	PAR	OCC	MCA
1 G	0.02 ± 0.01	0.03 ± 0.01	0.05 ± 0.02	0.02 ± 0.01
1.8 G	0.02 ± 0.01	0.03 ± 0.02	0.06 ± 0.03	0.02 ± 0.01
Statistical test	Ld.f2 model with Wald test			
*p *value	Factor *ROI*: <0.001			

Displayed are means ± standard deviation

*FRONT* frontal lobe, *PAR* parietal lobe, *OCC* occipital lobe, *MCA* middle cerebral artery

**Table 2:** Behavioural performance (error rate and reaction time) of 13 participants during normal gravity (1 G) and hypergravity (1.8 G) for the mental arithmetic task and the oddball paradigm

	Behavioural performance				
	Error rate [%]			Reaction time [ms]	
	Mental arithmetic task	Target sound	Standard sound	Mental arithmetic task	Target sound
1 G	17.73 ± 8.55	26.20 ± 20.77	3.69 ± 2.84	2332.21 ± 584	590.8 ± 82.12
1.8 G	19.73 ± 6.52	27.56 ± 16.28	4.58 ± 3.24	2214 ± 610.98	595.92 ± 78.12
Statistical test	RM ANOVA			RM ANOVA	
*p* value	0.94			0.06	
	n.s			n.s	

Displayed are means ± standard deviation

**Fig. 4:**
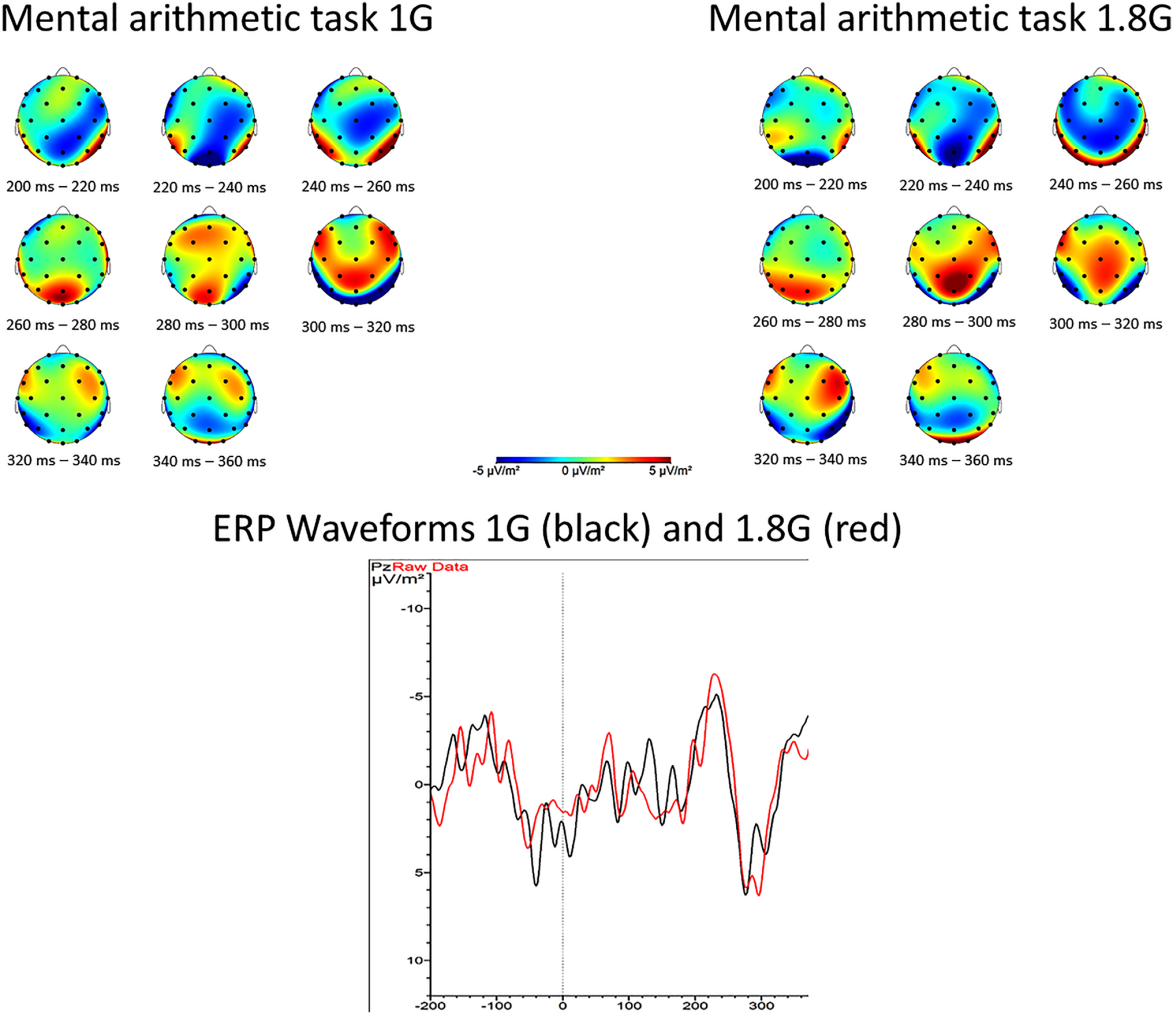
Top: topographical map showing the current source density (CSD) in steps of 20 ms over the scalp for 1 G (left) and 1.8 G (right). A clear positive peak around 260–280 ms after stimulus onset in the parietal scalp areas can be seen. Bottom: averaged event-related potentials (ERP) over all subjects for 1 G (black) and 1.8 G (red) provoked by the mental arithmetic task over Pz

**Fig. 5:**
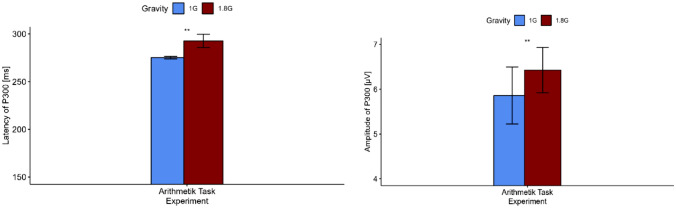
Event-related potentials amplitude and latency (*n* = 13) of P300 over Pz occurring for the mental arithmetic task in 1 G (blue) and 1.8 G (red). Displayed are means standard deviation. ^**^Marks *p* < 0.01

**Fig. 6:**
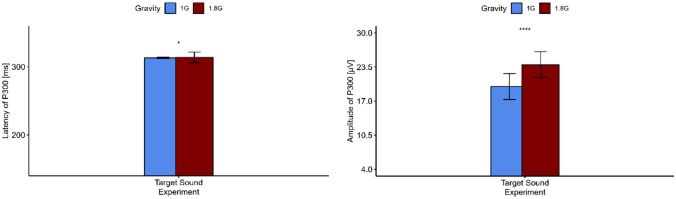
Event-related potentials amplitude and latency (*n* = 13) of P300 over Oz occurring for the target sound of the oddball task in 1 G (blue) and 1.8 G (red). Displayed are means ± standard deviation. *Marks *p* < 0.05, ****Marks *p* < 0.0001

Regarding the mental arithmetic task of the experiment, the P300 wave was highest over the Pz electrode and occurred significantly later in hypergravity (*V* = 0, *p* = 0.002) and with a higher amplitude (*t* = −3.67, *p* = 0.003) (s. Figs 4, 5 and Table 3). Regarding the target sound of the oddball task of the experiment, a P300 wave was visible over the occipital lobe under the electrode Oz. Like the P300 elicited by the mental arithmetic task, the P300 occurs later (*V* = 13, *p* = 0.022) and with a significantly higher amplitude in hypergravity (*V* = 0, *p* < 0.001) (s. Fig. 6; Table 3). The ld.f2 model with the Wald type (WTS) showed no interaction effect of the factor *gravity* and *task* regarding the latency of N200 (Statistic = 1.80, *p* = 0.18). However, there was a main effect of the factor *gravity* (Statistic = 11.36, *p* < 0.001) and *task* (Statistics = 204.887, *p* < 0.001) on the latency of N200.

**Table 3:** Amplitude and latency of event-related potentials N200 and P300 during normal gravity (1 G) and hypergravity (1.8 G) for the mental arithmetic task and the oddball paradigm

	Event-related potential			
	P300			
	Mental arithmetic task		Target sound (oddball paradigm)	
	Latency [ms]	Amplitude [µV]	Latency [ms]	Amplitude [µV]
1 G	275.23 ± 1.30	5.86 ± 0.64	313.23 ± 1.01	19.79 ± 2.48
1.8 G	292.77 ± 7.05	6.43 ± 0.51	313.85 ± 7.81	23.95 ± 2.46
Statistical test	Wilcoxon test	Paired *t*-test	Wilcoxon test	Wilcoxon test
*p* value	0.002	0.003	0.022	<0.001
	**	**	*	***

	N200			
	Oddball paradigm			
	Latency [ms]		Amplitude [µV]	
	Target sound	Standard sound	Target sound	Standard sound
1 G	203.08 ± 1.04	210.62 ± 2.50	−6.31 ± 0.3	−4.81 ± 0.21
1.8 G	201.69 ± 0.75	209.39 ± 3.67	−6.70 ± 0.73	−5.34 ± 0.27
Statistical test	Ld.f2 model with Wald test		Ld.f2 model with Wald test	
*p* value	Factor *Gravity*: <0.001 *Task*: <0.001		Factor *Gravity*: <0.001 *Task:* <0.001	

Displayed are means ± standard deviation

*Marks *p* < 0.05, **marks *p* < 0.01, ***marks *p* < 0.001

**Fig. 7:**
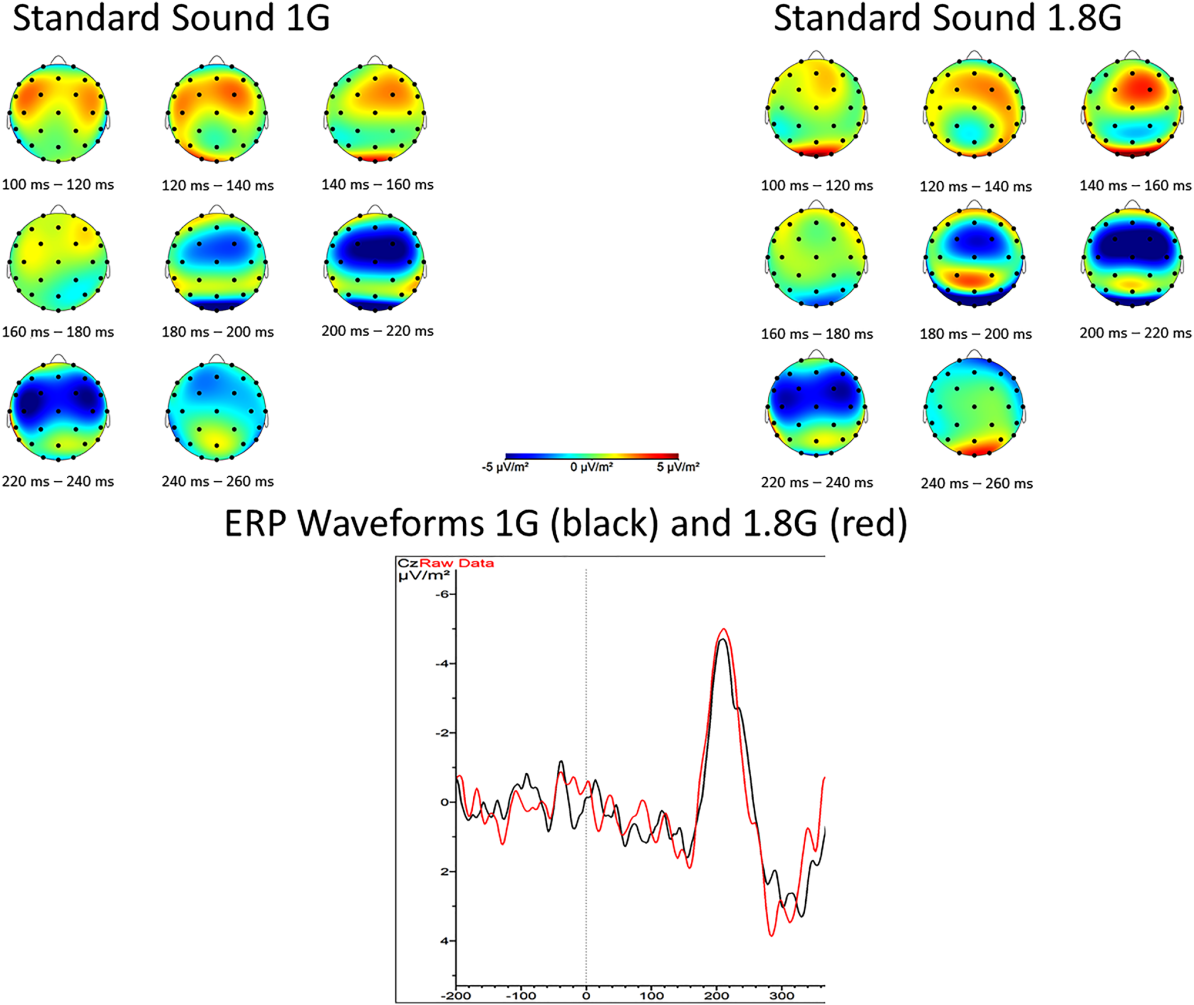
Top: topographical map showing the current source density (CSD) in steps of 20 ms over the scalp for 1 G (left) and 1.8 G (right). A clear negative peak around 200–220 ms after stimulus onset in centro-frontal scalp areas can be seen. Bottom: averaged event-related potentials (ERP) over all subjects for 1 G (black) and 1.8 G (red) provoked by the standard sound of the oddball task over Cz

**Fig. 8:**
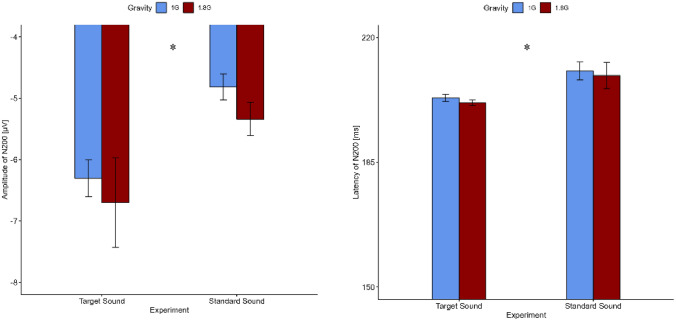
Event-related potentials amplitude and latency (*n* = 13) of N200 over Cz occurring for the target and standard sound of the oddball paradigm in 1 G (blue) and 1.8 G (red). Displayed are means ± standard deviation

The results for the amplitude of N200 showed no interaction effect (Statistic = 1.82, *p* = 0.18) but there are main effects of the factor *gravity* (Statistic = 34.896, *p* < 0.001) and *task* (Statistic = 11.36, *p* < 0.001) on the amplitude of N200 (s. Figs. 7, 8 and Table 3).

## Discussion

This study investigates the influence of short periods of hypergravity on electrocortical activity as well as neurocognitive performance. It is expected to contribute to a better understanding of neurophysiological changes under the influence of different gravity levels to ensure the safety of astronauts on upcoming space missions and, in the long term, to transfer the results to neurological rehabilitation.

No significant difference of 1 G and 1.8 G regarding electrocortical activity was recognisable in the different ROIs whereas the occipital lobe shows the greatest tendency with higher values in hypergravity. This region contains the visual cortex, which plays a major role in the identification of the primary task, as the mental arithmetic task appeared visually on the screen in front of the participants and they were asked to focus primarily on it. This tendency, in combination with the higher amplitude of P300 in hypergravity, which was elicited by the primary task and the target sound of the oddball paradigm and occurs only when the participant is actively engaged with the stimuli and making a response to it (Snyder and Hillyard 1976), the hypothesis of Klein et al. (2019) could be supported. Here the authors explained the significantly lower values of electrocortical activity in weightlessness with an increase in intracranial pressure, which leads to a slightly depolarised resting membrane potential of neurons (Kohn 2013; Wollseiffen et al. 2019). In hypergravity, haemodynamic alterations also occur, but instead of floating to the upper part of the body, as it happens in weightlessness, in hypergravity, there is a shift to the lower part of the body (Klein et al. 2020). There is supposedly no increase in intracranial pressure and no depolarisation of the resting membrane potential of nerve cells in hypergravity, as studies have already reported from weightlessness (Hanke and Schule 1993; Sieber et al. 2014, 2016). It is possible to speculate that the higher gravity condition could lead to a reversed physiological phenomenon and may decrease the resting membrane potential, making it harder to reach the threshold for triggering an action potential. Consequently, the higher values in hypergravity may serve as an indicator for the robustness of the model of Kohn et al. (Kohn and Ritzmann 2018).

The behavioural parameters error rate and reaction time showed no differences between 1.8 G and 1 G. Compared to Wollseiffen et al. (2019), who proved a positive effect of weightlessness on behavioural parameters, exposure to hypergravity does not seem to make any difference to normal gravity and could thus be declared as a non-negative influence.

The N200 wave was mainly triggered by the oddball paradigm of the experiment and was located in the fronto-central area under the electrode Cz. In terms of temporal occurrence according to the oddball paradigm, the lf.2D function with the Wald test showed no significant interaction effect of the within-group factors *gravity* and *task*. Nevertheless, the main effect of the factor *gravity* revealed an earlier occurrence in hypergravity. The significant differences in the latency of both event-related potentials, N200 and P300, can be related to the low standard deviations (s. Table 3). This in turn is in line with the reliable latency of ERP’s described in the literature (Deetjen et al. 2005; Patel and Azzam 2005), but in return ensures that even the smallest differences are already considered as significant.

Similar to the P300 wave, there was no interaction effect for the amplitude of N200. The within-group factors *gravity* and *task* individually, however, showed a highly significant influence on the amplitude with a larger amplitude triggered by the target sound than by the standard sound, which is consistent with the literature describing an unexpected event as the trigger for an N200 wave (Patel and Azzam 2005). The standard sound itself triggered a higher amplitude in hypergravity, which could also be explained by the model of Kohn et al. (Kohn and Ritzmann 2018).

Contrary to our assumption, hypergravity did not generally negatively influence electrocortical activity and neurocognitive performance. The behavioural performance of the participants does not seem to be negatively affected by hypergravity. Nevertheless, the event-related potentials showed significantly increased amplitude in hypergravity, which could underline the results of Wollseiffen et al. (2019) and Klein et al. (2019), who found significantly lower parameters in reduced gravity, which in this case is 1 G compared to 1.8 G. They both explain their findings on a physiological level with the model by Kohn et al. (Kohn and Ritzmann 2018), which reports changes in neural communication in weightlessness. Due to an increase of intracranial pressure due to the fluid shift in weightlessness (Lawley et al. 2017), the lateral pressure on the membrane of the neuronal cells increases and leads to a reduced open state probability of ion channels. This phenomenon then results in a slightly depolarized resting membrane potential up to 3 mV (Kohn and Ritzmann 2018).

In general, no negative effect of short phases of hypergravity could be found within this study. Besides this, the findings provide support for the physiological model that explains physiological changes in the central nervous system depending on different levels of gravity. No changes could be found within the frontal cortex, which is mainly responsible for executive functions and primarily targeted when solving a neurocognitive task. It is plausible to propose, that the relationship between the parameters and gravity may not follow a linear pattern but be more dependent on a threshold. This hypothesis aligns with the possible adaption of a model of cell communication proposed by Kohn et al. (Kohn and Ritzmann 2018).

The participants in this study were only exposed to hypergravity in (repeated) 20–30 s intervals. It is, therefore, questionable to what extent the exclusively short intervals have an influence on the results. Further studies should investigate longer periods of hypergravity to underline the findings, and investigations should be carried out which allows a more precise grading of gravity levels to find a possible existing threshold. This is of utmost importance regarding the plans of national and international space agencies for upcoming space missions to the Moon and Mars with a gravity of 0.16 G and 0.38 G, respectively.

## Data Availability

The datasets generated and analysed during the current study are available from the corresponding author on reasonable request.
